# Comparison of 23 Gauge Transconjunctival releasable Suture Vitrectomy with standard 20 gauge Vitrectomy

**DOI:** 10.12669/pjms.342.14234

**Published:** 2018

**Authors:** Haroon Tayyab, Asad Aslam Khan, Muhammad Ali Ayaz Sadiq, Irfan Karamat

**Affiliations:** 1 Dr. Haroon Tayyab, FCPS (Ophth), FCPS (Vitreoretinal Ophthalmology), FICO. Department of Ophthalmology, King Edward Medical College Mayo Hospital, Lahore, Pakistan; 2 Prof. Asad Aslam Khan, MS, PhD. Department of Ophthalmology, King Edward Medical College Mayo Hospital, Lahore, Pakistan; 3 Dr. Muhammad Ali Ayaz Sadiq, MD, FCPS, FAAPOS. Department of Ophthalmology, King Edward Medical College Mayo Hospital, Lahore, Pakistan; 4 Dr. Irfan Karamat, FCPS, MRCS. Department of Ophthalmology, King Edward Medical College Mayo Hospital, Lahore, Pakistan

**Keywords:** Intraocular pressure, Pars plana vitrectomy, Rhegmatogenous retinal detachment, Silicone oil

## Abstract

**Objective:**

To compare effectiveness of releasable transconjunctival sutures in 23 gauge vitrectomy and standard 20 gauge vitrectomy.

**Methods:**

This prospective comparative study was conducted in Department of Vitreoretinal Surgery, Al Ehsan Eye Hospital, Lahore from June 2016 to March 2017. It included 84 patients in total (Group-A: 42 patients underwent 23 gauge releasable suture vitrectomy; Group-B: 42 patients who underwent standard 20 gauge vitrectomy). Pre operative and post operative best corrected visual acuity, surgical duration, pre and post operative intraocular pressure and complication profile was compared between two groups.

**Results:**

The leading cause for vitrectomy was vitreous haemorrhage. (Group-A; n=15 ;35.71%; Group-B; n=17; 40.47%). There was statistically significant improvement in preoperative and postoperative BCVA in both groups (Group A: P-value < 0.05; Group B P-value < 0.05) but there was no significant difference in post operative BCVA between two groups at 3 months (P-value > 0.05). Surgical time for 23G vitrectomy Group was statistically less than 20 G vitrectomy Group (51 +/-18 minutes for Group-A versus 78 +/- 13 minutes for Group-B; p-value < 0.05). Visual analog score for pain / discomfort was also significantly less for Group-A than Group-B. There was no significant difference in intraocular pressures between the two groups.

**Conclusions:**

Releasable suture technique for small gauge vitrectomy is a safe and easily adaptable technique that has certain significant advantages over 20G absorbable suture vitrectomy.

## INTRODUCTION

The introduction of small gauge vitrectomy has given vitreoretinal surgeons an alternative of a less invasive surgical option when compared to traditional 20 gauge (20G) pars plana vitrectomy.^1^ Earliest small gauge vitrectomy systems utilized 23 gauge (23G) trocars, cannulas and instruments and was first introduced by Claus Eckhardt.^2^ Now, 23G vitrectomy is considered standard option for most advanced retinal surgeries.^2^ The main aim of smaller gauge vitrectomy systems is to reduce operative and recovery times, able to perform more delicate and intricate manoeuvres during surgery and avoid the complications known with larger gauge instruments. This quest has led to introduction of further small gauge instruments like 25 and 27 gauge. This has been possible because of innovations like more powerful light sources, rigid materials for small gauge instruments and more efficient vitrectomy machines with better fluidics and controls.^3^,^4^

With experience we have come to know that small gauge vitrectomy is not completely without complications. One of the most common complications noted in immediate post operative period of 23G vitrectomy is hypotony.^5^ This especially happens when partially competent sclerotomy sites are left suturesless after completion of vitrectomy. Other complications of suture less 23G vitrectomy include choroidal detachments and subconjunctival silicone oil in early post operative period.^5^,^6^ Some histological studies have confirmed the presence of subconjunctival silicone oil in upto 30% of cases that cannot be detected on routine slit lamp biomicroscopy.^7^ Another case series showed that 8-10% patients may have subconjunctival silicone oil bubble after undergoing sutures less 23G vitrectomy.^8^,^9^ In the light of these observations, it has been generally recommended not to leave 23G sclerotomy sites without sutures when silicone oil is used as a tamponading agent.^6^ On the other hand, application of sutures at sclerotomy site has been associated with more patient discomfort, higher surgically induced astigmatism, inflammation, suture granuloma formation and delayed visual recovery.^10^-^12^

Based on these observations, we decided to perform a novel way of securing sclerotomy sites with vicryl sutures that can be removed on the first post operative day at slit lamp. We applied a shoe lace styled releasable vicryl suture to combat the problems discussed above. Although there have been studies where effectiveness and safety of releasable vicryl sutures has been documented, we wanted to perform this technique in local population since local literature lacks the evidence of effectiveness of this technique.^13^,^14^ Given the simplicity of technique, we performed the following study to evaluate its effectiveness.

## METHODS

We conducted this prospective interventional case series in Department of Vitreoretinal surgery, Al Ehsan Eye Hospital, Lahore from June 2016 to March 2017. We included a total of 84 patients which were assigned into two groups A and B by simple random sampling method. Group-A underwent 23G vitrectomy with releasable sutures whereas Group-B underwent 20G vitrectomy with sutured sclerotomies. All patients were followed for three months after surgery. An informed consent was obtained from all participants of this study where potential side effects, benefits and nature of intervention were explained to the patients. Hospital ethical committee permission was duly sought before commencing this study. All patients who required phacoemulsification at time of vitrectomy, history of previous vitreoretinal surgery, known case of glaucoma, previous trabculectomy or who needed additional buckle were not included in this study.

The procedures were considered safe if there were no events of hypotony after removal of sutures. The efficacy of procedures was established through analysing post operative variables like BCVA, IOP and patient comfort (measured with help of visual analogue scale.

Post operatively, we measured intraocular pressure (IOP), best corrected visual acuity and post operative patient discomfort using visual analogue scale (VAS). Visual Analogue Scale is a measurement instrument that tries to measure a characteristic or attitude that is believed to range across a continuum of values and cannot easily be directly measured. It is often used in epidemiological and clinical research to measure the intensity or frequency of various symptoms. This visual analogue scale ranged from 0-4 with four showing maximum ocular discomfort.

For both Groups A and B, Faros Vitrectomy System (Oertli Instrumente AG, Switzerland) was used. We used Oculus BIOM 2 with Oculus SDI Inverter 2 (OCULUS Surgical, Inc. Port St. Lucie, USA) for retinal visualisation. Vitra Multispot 532nm green laser (Quantel Medical, Bozeman - US) was used for endolaser. Other vitreoretinal surgical adjuncts used in our study were RS-OIL ECS Silicone oil 1.000 cS, GOT Multi SF6 - pure sulphur hexafluoride, GOT Multi C3F8 - pure octafluoropropane gas and HPF10 high purity perfluorodecalin (AL.CHI.MI.A. SRL - Viale Austria).

In 23G vitrectomy group all sclerotomies were made using trocar cannula set provided by Faros Vitrectomy System (Oertli Instrumente AG, Switzerland). All incisions were made at 3.5mm from the limbus with oblique entry in sclera at an angle of approximately 10-15 degrees. Standard vitrectomy was performed and ports were closed using 7-0 Vicryl sutures (Ethicon Inc., Somerville, NJ, USA). We practiced a very low threshold for applying sutures whenever we felt that the sclerotomy site is incompetent. Application of suture was decided after we observed continued leakage of gas (air bubbles noted when eye surface was continuously irrigated under mild digital pressure with basic salt solution) even after rubbing posterior edge of sclerotomy for few seconds to allow self-sealing of wound. We applied sutures to all three ports where silicone oil was used a tamponade. All sutures were releasable in nature with a configuration of shoe lace for easy removal. We removed all sutures after one day at first post operative checkup using topical anaesthesia at slit lamp biomicroscope.

In case of 20G group, surgery was done after standard conjunctival peritomy and 20G straight scleral incision circumferential to limbus. All ports and conjunctiva was closed using 7-0 Vicryl suture at the end of procedure.

Best corrected visual acuity was recorded for all patients pre and post operatively but was not used in data analysis due to presence of intraocular gas in some eyes. We measured IOP using Goldmann applanation tonometer at one week after surgery; and patient discomfort was recorded using visual analogue scale at 1st, 2nd and 7th post op day. This visual analogue scale ranged from 0-4 with four showing maximum ocular discomfort. We used SPSS statistical software (version 20.0; SPSS Inc., Chicago, Illinois, USA) for data analysis. Numerical data was presented as mean +/- standard deviation. For other outcome variables, we used percentages, two-tailed paired *t*-test, Wilcoxon signed rank test, Mann Whitney’s *U*-test and p <0.05 was considered statistically significant.

## RESULTS

A total of 84 patients were included in this study that were divided into two equal groups A and B. The mean +/- standard deviation (SD) age of patients in Group A was 51.33 +/- 14.98 years where as that in Group B was 54.66 +/- 12.87 years. In Group A, 23/42 (54.7%) were females and 19/42 (45.23%) were males. In Group B, 28/42 (66.6%) were females where 14/42 (33.3%) were males. The most common aetiologies leading to pars plana vitrectomy in this study are shown in Table-I for both the groups. Leading cause of vitreous haemorrhage is shown in Table-II.

**Table-I:** Most common aetiology leading to pars plana vitrectomy in Group A & B.

	VH n (%)	RRD n (%)	TRD n (%)	VMI Pathologies n (%)	Dropped Nucleas / IO n (%)	Others n (%)
Group A	15 (35.7)	9 (21.4)	4 (9.5)	7 (16.6)	2 (4.7)	5 (11.9)
Group B	17 (40.4)	7 (16.6)	6 (14.3)	3 (7.1)	3 (7.1)	6 (14.3)

**Table-II:** Leading cause of vitreous hemorrhage in Group A & B.

	Diabetes Mellitus n (%)	Trauma n (%)	Post PVD n (%)	Eales Disease n (%)	RVO n (%)
Group A	9 (60)	2 (13.3)	2 (13.3)	1 (6.6)	1 (6.6)
Group B	11 (64.7)	2 (11.7)	1 (5.8)	1 (5.8)	2 (11.7)

In Group A, 6 (14.3%) out of 42 patients received gas as final intraocular tamponade where as in Group B, 8 (19%) out of 42 patients received gas. These patients were not included in the post operative best corrected visual acuity analysis due to presence of intraocular gas.

Best corrected visual acuity (BCVA) significantly improved pre and post operatively in both the groups. In Group-A, mean BCVA improved from 1.35 +/- 0.42 to 0.7 +/- 0.31 (P-value < 0.05). In Group-B, mean BCVA improved from 1.29 +/- 0.22 to 0.76 +/- 0.18 (P-value < 0.05). There was no statistically significant difference in the mean post operative BCVA between two groups.

There was no statistically significant difference in the pre operative intraocular pressure (IOP) between two groups (Group-A 14.05 mmHg +/- 3.55; Group-B 14.83 mmHg +/- 3.47; pvalue > 0.05). At seven days follow up, there was no post operative difference in IOP within the Groups (Group-A 15.28 mmHg +/- 3.24; p-value > 0.05 - Group-B 15.26 mmHg +/- 3.98; p-value > 0.05) and between the groups (Group-A 15.28 mmHg +/- 3.24; Group-B 15.26 mmHg +/- 3.98; p-value > 0.05).

Post operative pain score at 1st day, 2nd day and 7th day post op are given in. Table-III. There was significant difference in the discomfort reported by patients on day one in Groups A and B (p-value < 0.05) but this difference became non significant on post op day 7 (p-value = 0.43).

**Table-III:** Visual analog pain score for Group A and B at 1st day, 2nd day and 7th day post op.

	Day 1 Mean +/-SD	Day 2 Mean +/-SD	Day 7 Mean +/-SD
Group A	0.6 +/- 0.33	0.4 +/- 0.39	0.31 +/- 0.21
Group B	1.3 +/- 0.45	0.7 +/- 0.3	0.5 +/- 0.37
p- value	0.03	0.12	0.43

All sclerotomies in Group-B were secured with 7 0 Vicryl sutures. The average number of sutures required in Group-A (23 G group) were 51 out of 126 sclerotomies. Patients receiving silicone oil required more frequent suture application at sclerotomy site than those patients who received intravitreal gas or basic salt solution at the end of surgery. All sutures applied in 23 G group (Group-A) were removed on 1st post op day at slit lamp under topical anaesthesia and we do not report any untoward incident while performing this manoeuvre. No patient developed hypotony in case where releasable suture was removed in the early post operative period. There were no untoward complications reported in both the study groups.

Our surgical time for 23 G vitrectomy was considerably less as compared to 20 G vitrectomy. The mean surgical time for 23 G vitrectomy was 51 +/-18 minutes where as that of 20 G vitrectomy was 78 +/- 13 minutes (p-value < 0.05).

## DISCUSSION

We used releasable transconjunctival sutures for 23G vitrectomy in Group-A to assess their safety and efficacy in terms immediate post operative recovery. The main advantage of this technique is that removal of sutures is easy and pain free, avoid suture related complications and prevent post operative hypotony which may be associated with suture less vitrectomy. In our study, the average duration before which we removed these releasable sutures ranged from 19 to 24 hours post operatively. The other Group underwent standard 20G vitrectomy where all 3 ports were secured with 7 0 vicryl suture at the end of surgery.

It has been noted in various studies that scleral sutures become necessary in case of incompetent sclerotomies even after wound massage when undergoing 23G vitrectomy.^15^ These sutures may lead to higher incidence of post operative astigmatism.^16^ This surgically induced astigmatism is temporary and usually returns back to preoperative values at four months post op.^17^,^18^ It was also reported by Park and Shao in separate studies that surgically induced astigmatism was considerably less for 23 G vitrectomy as compared to 20 G vitrectomy.^19^,^20^ Our surgical time was also comparable to other similar studies conducted. In one study, Kim et al. reported significantly shorter surgical duration for 23G vitrectomy as compared to 20G. They reported that the mean operation time was 88.5 ± 20.1 in the 23G vitrectomy group and 102.1 ± 23.1 minutes in the 20G conventional group, respectively (*p*=0.01).^13^ This result was similar to results reported in our study. The difference in the surgical duration was mainly because of opening and closing time required with 20G vitrectomy despite that 20G vitrectomy is quicker once we exclude the opening and closing time required before starting vitrectomy.^13^

We did not experience any events of immediate or delayed post operative hypotony after suture removal in our study. Various studies have reported differing post operative hypotony rates in case of small gauge suturesless vitrectomies.^21^ Despite improvements in trocar cannula designs and our better understanding of scleral entry angles, scleral wound incompetence is encountered despite massaging of wound at the end of vitrectomy. Such port leaks may lead to hypotony, infections, choroidal detachments and loss of effective tamponade.^22^,^23^ Surgeons are now more keen to apply sutures to visibly incompetent sclerotomies even with smaller gauge vitrectomies to avoid these potential complications. However sutures may lead to irritation, foreign body sensation, suture related granuloma and rarely suture site infections.^24^ To avoid all this, there have been few studies evaluating the results and safety of releasable sutures for small gauge vitrectomies. As such, our results are comparable to Song et al. regarding post operative IOP in immediate and intermediate post operative period when evaluating the safety of early releasable sutures.^25^

Another interesting finding in our study was that total number of sutures required in 23G group was more in cases which required complete vitrectomy (retinal detachment, vitreous haemorrhage etc.) as compared to those requiring partial vitrectomy (macular hole, epiretinal membrane etc). This observation is comparable to another such study conducted recently by Kim et al.^13^

### Limitations of the study

Short follow up period, small sample size and surgery performed for variable retinal pathologies. However, the strong points about our study are that surgery was performed by a single surgeon under uniform operating conditions, prospective study design and careful patient selection before surrey.

In our opinion, the releasable suture technique can be widely adopted since it provides the dual benefit of sutured ports (thus avoiding hypotony) and no suture related complications. Although we have reported similar short term visual results between both the groups, the 23 G group with releasable suture vitrectomy did report better patient comfort, no post operative hypotony and no suture related complications. Although more studies need to be conducted regarding this technique, we safely recommend use of releasable suture technique for small gauge vitrectomy.

### Authors` Contribution

**HT** primary surgeon, designed study and collected data.

**AAK, MAAS** did review of literature and statistical analysis.

**IK** did review, final approval of manuscript and bibliography.
